# Robot-Assisted Kyphoplasty versus Fluoroscopy-Assisted Kyphoplasty: A Meta-Analysis of Postoperative Outcomes

**DOI:** 10.3390/medicina59040662

**Published:** 2023-03-27

**Authors:** Yu Chang, Wei-Cheng Chen, Kuan-Yu Chi, Abel Po-Hao Huang, Shang-Wun Jhang, Li-Wei Sun, Chien-Min Chen

**Affiliations:** 1 Section of Neurosurgery, Department of Surgery, National Cheng Kung University Hospital, College of Medicine, National Cheng Kung University, Tainan 704, Taiwan; yuchang1112359@gmail.com; 2 Department of Orthopedics, Shuang Ho Hospital, Taipei Medical University, Taipei 235, Taiwan; p830113@gmail.com; 3 Department of Education, Center for Evidence-Based Medicine, Taipei Medical University Hospital, Taipei 110, Taiwan; kuanyuhippo@gmail.com; 4 Division of Neurosurgery, Department of Surgery, National Taiwan University Hospital, National Taiwan University College of Medicine, Taipei 100, Taiwan; how.how0622@gmail.com; 5 Division of Neurosurgery, Department of Surgery, Changhua Christian Hospital, Changhua 500, Taiwan; 133393@cch.org.tw (S.-W.J.); 182034@cch.org.tw (L.-W.S.); 6 College of Nursing and Health Sciences, Dayeh University, Changhua 515, Taiwan; 7 Department of Leisure Industry Management, National Chin-Yi University of Technology, Taichung 433, Taiwan

**Keywords:** robot-assisted kyphoplasty, fluoroscopy-assisted kyphoplasty, meta-analysis

## Abstract

Osteoporotic vertebral compression fractures are the most common manifestation of osteoporosis. Percutaneous kyphoplasty (PKP) can lead to both pain improvement and correction of kyphosis secondary to collapsed vertebral bodies. Robot-assisted (RA) PKP has been reported to provide better vertebral body fracture correction than conventional fluoroscopy-assisted (FA) PKP. The aim of this meta-analysis is to compare clinical outcomes of RA PKP versus FA PKP. The Pubmed, Embase, and MEDLINE electronic databases were searched from January 1900 to December 2022, with no language restrictions for relevant articles. We extracted the preoperative and postoperative mean pain score and standard deviation from the included studies and pooled them using an inverse variance method. Statistical analyses were performed using functions available in the metafor package in R software. The results of this meta-analysis were summarized with weighted mean differences (WMDs). Our search strategy identified 181 references from the Pubmed, Embase, and MEDLINE electronic databases. We excluded duplicates and irrelevant references, after screening titles and abstracts. The remaining 12 studies were retrieved for full-text review, and, finally, we included five retrospective cohort studies from 2015 to 2021, comprising 223 patients undergoing RA PKP and 246 patients undergoing FA PKP. No difference was found in subgroup analysis based on the timing of postoperative pain assessment, despite the overall estimate of postoperative pain indicating a significant difference between the RA PKP and FA PKP groups (WMD, −0.22; 95% CI, −0.39 to −0.05). The long-term pain assessment revealed a significantly lower VAS in the RA PKP group than the FA PKP group at six months postoperatively (WMD, −0.15; 95% CI, −0.30 to −0.01), but no difference between the subgroups at three (WMD, 0.06; 95% CI, −0.41 to −0.54) and twelve months (WMD, −0.10; 95% CI, −0.50 to 0.30) postoperatively. Our meta-analysis revealed no significant difference in postoperative pain between RA PKP and FA PKP. Patients undergoing RA PKP had better pain improvement compared to FA PKP at 6 months postoperatively. However, further studies focusing on long-term outcomes in patients undergoing RA PKP are warranted to clarify its benefit, given the small number of included studies.

## 1. Introduction

Osteoporotic vertebral compression fractures (OCVFs) are the most common manifestation of osteoporosis, affecting millions of people worldwide, especially postmenopausal women and elderly individuals. OCVFs result from the deterioration of the bone structure, which leads to a weakened vertebral body and an increased risk of fracture, even from minor trauma or loading. These fractures can cause significant morbidity and can result in chronic back pain, height loss, spinal deformity, and impaired quality of life. Additionally, OCVFs are associated with an increased risk of future vertebral and non-vertebral fractures, leading to higher healthcare costs and increased mortality rates. Despite the high prevalence and impact of OCVFs, they are often underdiagnosed and undertreated, and many patients do not receive appropriate care, including pain management, fracture prevention, and rehabilitation. Therefore, there is a need for better understanding, prevention, and management of OCVFs to improve patients’ outcomes, reduce healthcare costs, and enhance the quality of life of individuals with osteoporosis [1].

Vertebral fractures of the thoracic and lumbar spine are a significant public health concern, accounting for approximately 700,000 of the 1.5 million osteoporotic fractures occurring annually in the United States alone [2]. These fractures can lead to chronic pain, decreased mobility, kyphosis, and an overall reduction in quality of life. Furthermore, they can lead to increased morbidity and mortality, particularly in elderly patients. In addition to the physical impact, vertebral fractures also have significant economic implications, with estimates of direct and indirect costs related to osteoporotic fractures exceeding USD 19 billion annually in the United States alone. Given the significant burden of osteoporotic vertebral fractures, there is a critical need to develop effective treatment and management strategies to reduce their incidence and impact. The increased incidence of OCVFs worldwide, paired with increased life expectancy, especially in those aged over 65 years, highlights the need for effective prevention and treatment strategies to address this growing health issue [3].

The most commonly seen symptom of OCVF is back pain [1]. In addition to the symptoms mentioned, OCVFs can also lead to an increased risk of falls, which can result in further fractures and other injuries. Moreover, untreated OCVFs can cause significant physical and emotional stress, as individuals may experience decreased mobility, increased dependence on others, and a reduced ability to participate in daily activities [1,3]. It is essential to promptly diagnose and manage OCVFs to minimize the impact on patients’ quality of life and to prevent potential complications. Therefore, early recognition and appropriate management of OCVFs are crucial to reduce morbidity, mortality, and healthcare costs associated with this condition. Further intervention for persistent back pain is indicated for those with OCVFs exhibiting a poor response to conservative medical treatment, including analgesia, antiosteoporosis treatment, and bed rest [1].

The two mainstay interventions for OCVFs are percutaneous vertebroplasty (PVP) and percutaneous kyphoplasty (PKP) [4]. PVP is performed by injecting bone cement into a fractured vertebral body, and evidence showed postoperative pain improvement following PVP [4]. PKP, compared to PVP, is performed by inserting an expandable device into the fracture vertebral body, and the cavity created by the device is injected with bone cement [4]. PKP can lead to both pain improvement and correction of kyphosis secondary to collapsed vertebral bodies [5]. In recent years, there has been ongoing debate about the effectiveness of PVP and PKP in the treatment of OCVFs, with some studies showing conflicting results. While both procedures have been found to be effective in reducing pain and restoring vertebral body height, their long-term outcomes are still a topic of discussion. Some researchers have suggested that PKP may provide superior results in terms of pain relief and restoration of vertebral body height, while others argue that the difference between the two procedures is negligible. It is also important to note that both PVP and PKP carry a risk of complications, including cement leakage, pulmonary embolism, and infection, and these risks should be carefully considered when deciding which is the most appropriate intervention for a given patient. Overall, further research is needed to determine the optimal management approach for OCVFs and to develop more effective and safer treatment options for this condition.

PKP is a minimally invasive procedure, but as with any medical intervention, it is not entirely without risk [6]. Puncture injury can occur during the procedure, particularly if the needle used to access the vertebral body inadvertently punctures adjacent structures, such as nerves or blood vessels. Another potential complication is bone cement leakage, which can occur when the cement used to stabilize the vertebral body leaks into the surrounding tissues, leading to nerve irritation or compression, pulmonary embolism, or other adverse events [6,7]. While the incidence of these complications is generally low, clinicians should be vigilant in monitoring patients for signs of complications after PKP and take appropriate steps to manage any adverse events that may arise. Traditionally, surgeons use a fluoroscopy-based device during the procedure to guide and assist PKP surgery [4]. Robots have been emerging to assist spine surgeries [8], and robot-assisted (RA) PKP reduces radiation exposure of the surgeon and the operating room staff [9] and provides better vertebral body fracture correction than conventional fluoroscopy-assisted (FA) PKP [10]. Several studies compared the radiographic outcomes and procedure times between RA PKP and FA PKP [11,12,13]. Additionally, studies investigated pain improvement following RA PKP [10,12], which is the most important outcome, because patients opt for PKP due to refractory pain. This study aimed to perform a systematic review and meta-analysis of the summarized current literature on the clinical outcomes of RA PKP versus FA PKP. By synthesizing and analyzing the available evidence from published studies, this study aimed to provide clinicians with reliable and up-to-date information to assist in clinical decision making regarding the selection of the most effective and safe treatment option for OCVFs. Ultimately, the goal of this study was to improve patient outcomes and quality of life by providing evidence-based recommendations for the management of OCVFs.

## 2. Methods

The systematic review and meta-analysis were conducted based on the Cochrane Handbook for Systematic Reviews and Interventions [14], and results were reported following the Preferred Reporting Items for Systematic Reviews and Meta-Analyses (PRISMA) (eMethod 1 in the Supplementary Materials).

### 2.1. Study Selection

The Pubmed, Embase, and MEDLINE electronic databases were searched from January 1900 to December 2022, with no language restrictions for relevant articles. The reference lists of all the included studies were also screened for potentially relevant articles that were not identified in the electronic search. The selection process for inclusion of studies was conducted in two stages: a title and abstract screening, followed by a full-text review. The details of the search process, including the search terms and search strings, are presented in eMethod2, which can be found in the Supplementary Materials. Two investigators (Y.C. and K.-Y.C.) independently performed the search to identify relevant studies for inclusion, with discrepancies resolved by either reaching a consensus or consulting a senior reviewer (C.-M.C.).

### 2.2. Eligibility Criteria

Articles meeting the following criteria were included: (1) randomized controlled trials, prospective and/or retrospective cohort and case-control studies (case reports, editorials, letters to the editor, review articles, and conference abstracts were excluded); (2) studies of adults undergoing PKP for vertebral body fractures; and (3) studies reporting comparative clinical outcomes (pain severity) of RA PKP versus FA PKP. In order to avoid duplication and ensure the highest quality of data in our meta-analysis, we only included the most complete reports of studies with an accumulated number of patients or increased follow-up durations.

### 2.3. Data Extraction

Two investigators (Y.C. and W.-C.C.) independently extracted the following data from eligible studies: first author’s last name, publication year, inclusion period, patient number, preoperative and postoperative pain score, and rate of cement leakage. The extracted data were entered into a pre-designed data extraction form in a standardized manner. Any discrepancies in the extracted data were resolved by discussion between the two investigators or by consulting a senior reviewer (C.-M.C.).

### 2.4. Quality Assessment

Critical appraisal of the included studies was performed to evaluate the quality of evidence and to determine the strength of the recommendations. The Risk of Bias in Non-randomized Studies of Interventions (ROBINS-I) [15] tool was chosen for critical appraisal of the included studies because it is a validated and widely accepted tool that provides a comprehensive evaluation of the risk of bias in non-randomized studies. The tool assesses bias across seven domains: confounding, selection of participants, classification of interventions, deviations from intended interventions, missing data, measurement of outcomes, and selection of the reported result. Two investigators (Y.C. and W.-C.C.) who independently assessed the studies were trained in the use of the ROBINS-I tool, and any disagreements between them were resolved by discussion or by consulting with a third investigator who had extensive experience in systematic reviews and meta-analyses (K.-Y.C.).

### 2.5. Statistical Analysis

Statistical analyses were performed using functions available in the metafor package in R software (eMethod 3). The metafor package includes various methods for estimating effect sizes, pooling effect sizes across studies, assessing heterogeneity, and performing sensitivity analyses to assess the robustness of the results. The package also provides options for generating forest plots, funnel plots, and other graphical representations of the data, which can aid in the interpretation and communication of the results [16]. The clinical outcomes of interest are back pain severity and improvement after PKP. We extracted the preoperative and postoperative mean pain score and standard deviation from the included studies and pooled them using an inverse variance method. The results were summarized with the weighted mean difference (WMD). The restricted maximum likelihood method [17] was used as a heterogeneity estimator to conduct random-effect meta-analyses because between-trial variance was inevitable. Effect sizes were presented with their corresponding 95% confidence intervals (CIs) with Hartung–Knapp–Sidik–Jonkman adjustment [18]. The assessment of heterogeneity is critical in meta-analysis because it affects the choice of statistical models and the interpretation of the results. Heterogeneity was assessed using I^2^ statistics proposed by Higgins and Thompson. It measures the percentage of total variation across studies due to heterogeneity rather than chance. An I^2^ value of less than 25% indicates low heterogeneity, which suggests that the studies are relatively consistent in their results. A value between 25% and 50% indicates moderate heterogeneity, which suggests that the studies have some degree of variability in their results. An I^2^ value of greater than 50% indicates high heterogeneity, which suggests that the studies are significantly different from each other [19]. 

## 3. Results

### 3.1. Study Selection

**Figure 1:**
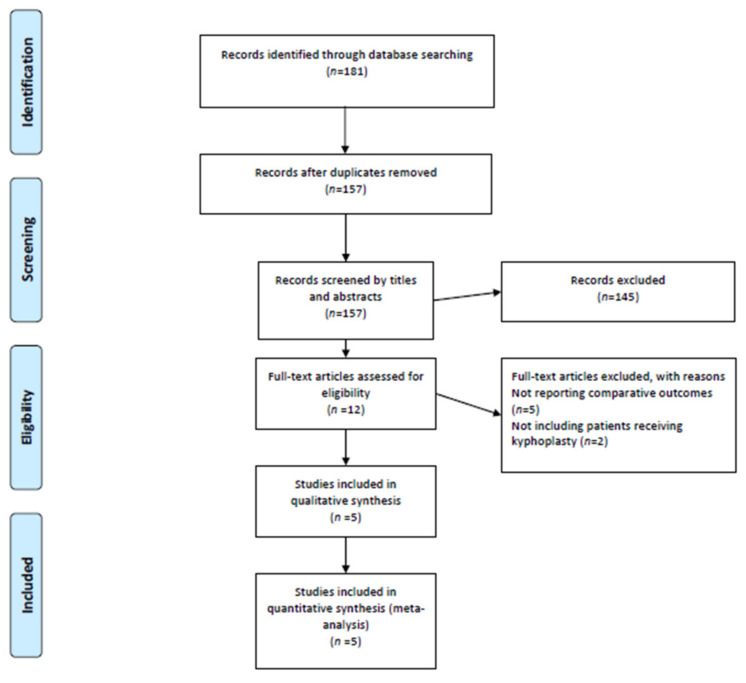
PRISMA flowchart diagram. We initially extracted 181 potential references. Screening the titles and abstracts yielded 12 full-text articles, the eligibility of which was assessed. Eventually, 5 studies were included for qualitative and quantitative syntheses. PRISMA, Preferred Reporting Items for Systematic Reviews and Meta-Analyses.

Our search strategy identified 181 references from the Pubmed, Embase, and MEDLINE electronic databases. We excluded duplicates (*n* = 24) and irrelevant references (*n* = 145) after screening titles and abstracts. The remaining 12 studies were retrieved for full-text review, 5 [10,11,12,13,20] of which were included in the review (Figure 1).

### 3.2. Study Characteristics 

As shown in Table 1, five retrospective cohort studies [10,11,12,13,20] were included from 2015 to 2021, comprising 223 patients undergoing RA PKP and 246 patients undergoing FA PKP. Four studies [11,12,13,20] included patients receiving PKP due to OCVFs and the other one included patients with traumatic vertebral body fractures [10]. The rate of cement leakage ranged from 1.23% to 46.2% in the RA PKP group and ranged from 1.53% to 90.6% in the FA PKP group.

### 3.3. Quality Assessment of the Included Studies

In this study, we assessed the quality of the included studies using the ROBINS-I tool. Our assessment revealed that two of the five studies had a critical risk of bias, mainly due to measurement of outcome, while the other three studies had a moderate risk of bias (Table 2).

### 3.4. Clinical Outcomes of RA PKP versus FA PKP

**Figure 2:**
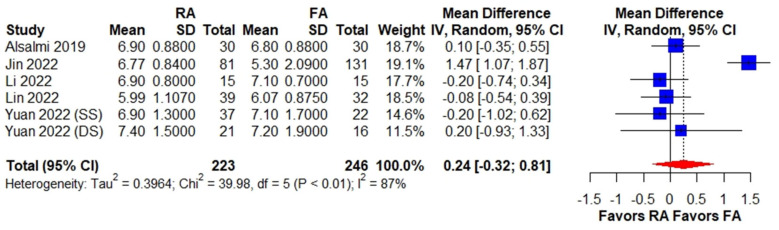
Forest plot of preoperative VAS among patients receiving RA PKP versus FA PKP. The forest plot demonstrates preoperative VAS among the RA PKP group versus the FA PKP group. The size of squares is proportional to the weight of each study. The result is summarized using weighted mean difference with corresponding 95% confidence interval (CI). Horizontal lines indicate the 95% CI of each study; the red diamond indicates the pooled estimate with 95% CI. CI, confidence interval, FA PKP, fluoroscopy-assisted kyphoplasty, RA PKP, robot-assisted kyphoplasty, VAS, visual analog scale [10,11,12,13,20].

Our meta-analysis revealed no significant difference in preoperative VAS between the RA PKP and FA PKP groups (WMD, 0.24; 95% CI, −0.32 to 0.81) (Figure 2).

**Figure 3:**
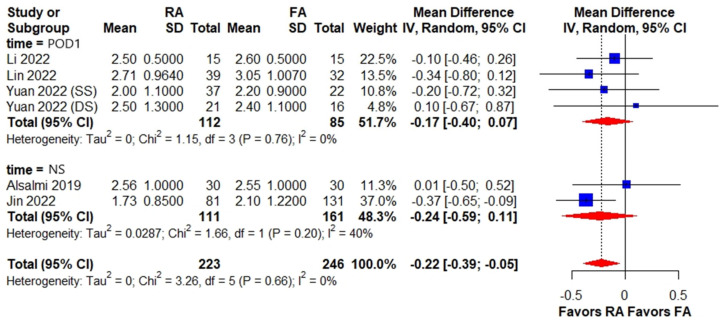
Forest plot of postoperative VAS among patients receiving RA PKP versus FA PKP. The forest plot demonstrates postoperative VAS among the RA PKP group versus the FA PKP group. The size of squares is proportional to the weight of each study. The result is summarized using weighted mean difference with corresponding 95% CI. Horizontal lines indicate the 95% CI of each study; red diamonds indicate the pooled estimate with 95% CI. CI, confidence interval, FA PKP, fluoroscopy-assisted kyphoplasty, NS, not-specified, POD1, postoperative day 1, RA PKP, robot-assisted kyphoplasty, VAS, visual analog scale [10,11,12,13,20].

**Figure 4:**
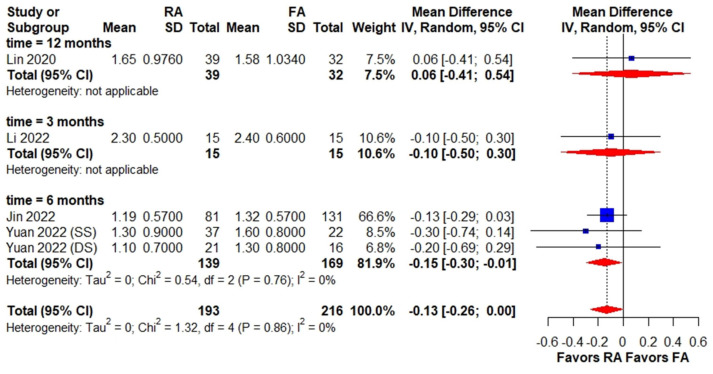
Forest plot of long-term VAS among patients receiving RA PKP versus FA PKP. The forest plot demonstrates long-term VAS among the RA PKP group versus the FA PKP group. The size of squares is proportional to the weight of each study. The result is summarized using weighted mean difference with corresponding 95% confidence interval (CI). Horizontal lines indicate the 95% CI of each study; red diamond indicates the pooled estimate with 95% CI. CI, confidence interval, FA PKP, fluoroscopy-assisted kyphoplasty, RA PKP, robot-assisted kyphoplasty, VAS, visual analog scale [11,12,13,20].

Three studies recorded postoperative day 1 VAS, which is a clinically relevant endpoint for assessing early postoperative pain. However, the other two studies did not have a clear definition of timing for postoperative pain assessment, which may have introduced some variability in the results. No difference was found in subgroup analysis based on the timing of postoperative pain assessment, despite the overall estimate of postoperative pain indicating a significant difference between the RA PKP and FA PKP groups (WMD, −0.22; 95% CI, −0.39 to −0.05) (Figure 3). The long-term pain assessment revealed a significantly lower VAS in the RA PKP group than the FA PKP group at six months postoperatively (WMD, −0.15; 95% CI, −0.30 to −0.01) but no difference between the subgroups at three (WMD, 0.06; 95% CI, −0.41 to −0.54) and twelve months (WMD, −0.10; 95% CI, −0.50 to 0.30) postoperatively (Figure 4).

## 4. Discussion

This systematic review aimed to compare the clinical outcomes of RA PKP and FA PKP procedures. Through a robust meta-analysis that employed a heterogeneity estimator and CI adjustment, no significant difference was found between the two groups in terms of postoperative pain. However, it is important to note that the benefit of long-term pain relief after RA PKP was observed only at six months postoperatively. On the other hand, a recently published systematic review [21] showed that RA PKP produced better results than FA PKP in terms of vertebral height and kyphosis angle. Despite this finding, our study found little difference in the postoperative pain improvement between the two groups. One possible explanation for this is the study by Feltes et al., which found that postoperative vertebral height did not interfere with pain improvement [22]. However, the methodological quality and patient characteristics of the studies included in our meta-analysis varied, which underscores the need for caution when interpreting our results. Larger sample sizes and more standardized methodologies are required to provide more definitive conclusions about the comparative effectiveness of these procedures. Despite the limitations, our study provides valuable insights into the clinical outcomes of RA PKP and FA PKP procedures and highlights the importance of considering factors, such as vertebral height and kyphosis angle, when assessing a patient’s overall improvement. Further research is needed to confirm our findings and improve the comparability of outcome measures across studies. Overall, our study contributes to the growing body of evidence on the effectiveness of different surgical techniques for the treatment of spinal conditions.

Compared to FA PKP, the higher precision of cement injection in RA PKP would be associated with a lower risk of leakage. In our review, we found a higher risk of cement leakage in the FA PKP group in all of the included studies. Theoretically, the expandable devices can be accurately positioned near the midline or fracture line during RA PKP, allowing the cement to be diffusely distributed along the midline of the vertebral body or fractured area, which can provide better pain relief [12]. Conversely, using traditional techniques for pedicle puncture, RA PKP often requires repeated adjustments to the puncture angle to ensure the correct trajectory. This can lead to increased damage to the surrounding muscles, fascia, and other soft tissues, ultimately resulting in insufficient pain relief after the operation [23]. However, postoperative pain is multifactorial and can be influenced by procedure time, the extent of the operation, and postoperative management [24]. Additionally, two studies did not report the timing of postoperative pain, which may also hinder an accurate assessment.

Our meta-analysis revealed no significant difference in preoperative VAS scores between the RA PKP and FA PKP group. It is important to note that preoperative pain levels can be affected by various factors, such as the duration of symptoms, the severity of the fracture, and the presence of comorbidities. The results of our meta-analysis for long-term pain improvement should be interpreted with caution because only four studies reported long-term outcomes with different follow-up times. The significant pain relief in the RA PKP group was only observed at six months postoperatively, while Lin et al. [12] revealed no difference twelve months postoperatively. Hence, further studies focusing on long-term outcomes in patients undergoing RA PKP are warranted to clarify its possible benefit.

Despite the promising findings of our meta-analysis, there were several limitations that should be taken into consideration when interpreting the results. First, the small number of included studies limits the generalizability of our findings and the statistical power of the analysis. Additionally, the retrospective design of the included studies increases the risk of bias due to confounding factors, such as differences in patient characteristics, treatment protocols, and outcome assessment methods. Second, the heterogeneity of the included studies in terms of patient baseline conditions, surgical techniques, and outcome measures may also have contributed to the variability in results across studies. This highlights the need for more standardized methodologies and larger-scale studies to enable more accurate comparisons between treatment options. Third, the timing of pain assessment varied across the included studies, which may have contributed to heterogeneity and limited the comparability of the results. Future studies should consider using consistent and standardized outcome measures and timing of assessments to enable more accurate comparisons between treatment options. Finally, most of the studies included in our meta-analysis were conducted in China, with only one study conducted in France. Therefore, our findings may not be generalizable to other populations, and further research is needed to explore the effectiveness of these procedures in other geographic regions and patient populations. Overall, while our meta-analysis provides some valuable insights into the comparative effectiveness of different surgical techniques for the treatment of certain conditions, further research is needed to address the limitations of our study and to provide more definitive conclusions.

## 5. Conclusions

The present meta-analysis provides valuable insights into the comparative clinical outcomes of RA PKP and FA PKP for the treatment of OCVFs. The finding of no significant difference in postoperative pain between the two procedures indicates that both techniques are similarly effective in relieving pain caused by OCVFs. However, our study shows that RA PKP may offer a benefit in terms of long-term pain relief at 6 months postoperatively, compared to FA PKP. While this finding is encouraging, it is important to note that the number of studies included in our analysis is relatively small, and further studies with larger sample sizes and longer follow-up periods are necessary to confirm these results. Additionally, future research should explore other important clinical outcomes, such as quality of life, functional status, and overall patient satisfaction, to better understand the comparative effectiveness of these procedures. Nonetheless, the results of this study provide clinicians and patients with valuable information regarding the potential benefits of RA PKP over FA PKP for the treatment of OCVFs.

## Figures and Tables

**Table 1:** Study characteristics.

Study	Study Design	Inclusion Period	Inclusion Criteria	Sample Size		Preoperative VAS, Mean (SD)		Postoperative VAS, Mean (SD)		Long-Term VAS, Mean (SD)		Cement Leaks Rate	
				RA	FA	RA	FA	RA	FA	RA	FA	RA	FA
Alsalmi 2019 [10]	RC	2015–2016	PKP for traumatic VBF	30	30	6.9 (0.88)	6.8 (0.88)	NA	NA	2.56 (1.0)	2.55 (1.1)	45.8%	71.9%
Jin 2022 [11]	RC	2020	PKP for OCVFs	81	131	6.77 (0.84)	5.30 (2.09)	1.19 (0.57)	1.32 (0.57)	1.73 (0.85)	2.10 (1.22)	1.23%	1.53%
Li 2022 [20]	RC	2019–2021	PKP for OCVFs	15	15	6.9 (0.8)	7.1 (0.7)	2.3 (0.5)	2.4 (0.6)	2.5 (0.5)	2.6 (0.5)	6.67%	60%
Lin 2022 [12]	RC	2018–2021	PKP for multi-segmental thoracolumbar OCVFs	39	32	5.99 (1.11)	6.07 (0.88)	1.65 (0.98)	1.58 (1.03)	2.71 (0.96)	3.05 (1.01)	46.2%	90.6
Yuan 2022 [13] (SS)	RC	2018–2019	PKP for OCVFs	37	22	6.9 (1.3)	7.1 (1.7)	1.3 (0.9)	1.6 (0.8)	2.0 (1.1)	2.2 (0.9)	8.1	27.3
Yuan 2022 [13] (DS)	RC	2018–2019	PKP for OCVFs	21	16	7.4 (1.5)	7.2 (1.9)	1.1 (0.7)	1.3 (0.8)	2.5(1.3)	2.4 (1.1)	9.5	28.1

FA, fluoroscopy-assisted, OCVF, osteoporotic vertebral compression fracture, PKP, percutaneous kyphoplasty, RA, robot-assisted, RC, retrospective cohort, SD, standard deviation, VAS, visual analogue scale, VBF, vertebral body fracture.

**Table 2:** Quality assessment of included studies.

Study	Bias Due to Confounding	Bias in Selection of Participants	Bias in Classification of Intervention	Bias Due to Deviation from Intended Intervention	Bias Due to Missing Data	Bias in Measurement of Outcome	Bias in the Selection of Selected Results	Overall Risk of Bias
Alsalmi 2019 [10]	Moderate	Low	Low	Low	Low	Critical	Low	Critical
Jin 2022 [11]	Moderate	Low	Low	Low	Low	Critical	Low	Critical
Li 2022 [20]	Moderate	Low	Low	Low	Low	Moderate	Low	Moderate
Lin 2022 [12]	Moderate	Low	Low	Low	Low	Moderate	Low	Moderate
Yuan 2022 [13]	Moderate	Low	Low	Low	Low	Moderate	Low	Moderate

## Data Availability

The detailed data of this study are available from the corresponding.

## Supplementary Materials

The following supporting information can be downloaded at: https://www.mdpi.com/article/10.3390/medicina59040662/s1, eMethod 1: Preferred Reporting Items for Systematic Reviews and Meta-Analyses (PRISMA) 2020 checklist; eMethod 2: Search strategy; eMethod 3: Data synthesis.
